# Wildlife Viruses: Impact on Human and Animal Health

**DOI:** 10.3390/v16081244

**Published:** 2024-08-02

**Authors:** Subir Sarker

**Affiliations:** 1 Biomedical Sciences and Molecular Biology, College of Public Health, Medical and Veterinary Sciences, James Cook University, Townsville, QLD 4811, Australia; subir.sarker@jcu.edu.au; 2 Australian Institute of Tropical Health and Medicine, James Cook University, Townsville, QLD 4811, Australia

In recent years, there has been a significant rise in the appearance of new viral infectious diseases among wildlife populations globally [1,2,3,4,5,6,7,8,9,10,11]. This trend presents an increasing threat to wildlife and contributes to the major diseases affecting human health [9]. Many of these emerging viral pathogens—including Ebola and Marburg viruses, human immunodeficiency viruses, Nipah virus, Sin Nombre virus, Hendra and Menangle viruses, West Nile virus, Middle East respiratory syndrome (MERS), and various subtypes of avian influenza—originate in wildlife and spill over into human hosts due to ecological, demographic, and socioeconomic changes [10]. The spread of severe acute respiratory syndrome coronavirus 2 (SARS-CoV-2) is a recent example that underscores the serious threat these viruses pose to both human populations and a wide range of wild animals, from amphibians to mammals [8]. Factors like habitat destruction, pollution, and international trade heighten the risk of viruses spreading to new hosts and causing disease.

The first paper in this Special Issue, authored by Athukorala et al. [12], delves into the intricate genetic diversity and evolutionary trajectories of avian adenoviruses. By leveraging complete genome sequences and partial DNA polymerase sequences sourced from the NCBI database, the study scrutinizes avian adenoviruses within the genera *Atadenovirus*, *Siadenovirus*, and *Aviadenovirus*. A pivotal discovery is the reclassification of the origin of *Atadenovirus* to an Australian native passerine bird, thereby challenging the previously held notion of a reptilian origin. Moreover, the study dispels the hypothesis that elevated AT content is a definitive characteristic of the *Atadenovirus* species. Through detailed phylogenetic analyses, the authors advocate for a reassessment of the origins of *Siadenovirus* and underscore the critical need for extensive research on avian adenoviruses in wild birds to enhance our comprehension of their evolutionary history. The paper further delineates the structural and genomic hallmarks of adenoviruses, including the identification of major capsid proteins and significant inter-generic variations in gene content. This investigation underscores the rapid advancements in adenovirus genomics, which are driven by cutting-edge molecular detection technologies such as next-generation sequencing. Collectively, the study furnishes a comprehensive genomic comparative analysis of adenoviruses, reexamines established evolutionary hypotheses, and proposes prospective avenues for future research in the realm of avian adenoviruses. The findings have profound implications for the taxonomy and comprehension of adenovirus diversity and evolutionary mechanisms, providing a foundation for subsequent explorations and discoveries in the field.

The second paper in this Special Issue, authored by Echeverry-Bonilla et al. [13], investigates the presence and genetic characterization of canine distemper virus (CDV) in the Crab-eating fox (*Cerdocyon thous*). CDV, a highly contagious morbillivirus, poses substantial conservation risks to both domestic and wild carnivores due to its ability to transmit across species. The study was prompted by the detection of three symptomatic Crab-eating foxes in distinct municipalities within the Tolima Department of Colombia in December 2021. These foxes underwent comprehensive physical assessments and diagnostic tests, including serological assays and reverse transcription polymerase chain reaction (RT-PCR) testing. The sequenced CDV strains from these foxes were phylogenetically classified within the South America/North America-4 lineage, which is closely related to strains with a previously reported presence in the United States. This study represents the first molecular identification of CDV in wildlife in Colombia, underscoring the virus’s potential for intercontinental dissemination via wildlife reservoirs. The findings emphasize the critical role of wildlife in the epidemiology of CDV, particularly at the interface between wildlife and domestic animals. Understanding these transmission patterns is essential for developing effective CDV prevention and control strategies, especially in regions with frequent wildlife–domestic animal interactions. This research underscores the urgent need for continuous surveillance and comprehensive molecular studies to mitigate the impact of this multi-host pathogen on both domestic and wild carnivore populations, ensuring robust conservation and public health measures.

The third paper in this Special Issue, authored by Savini et al. [14], presents a thorough investigation of West Nile virus (WNV) and Usutu virus (USUV) surveillance in avian species, focusing on raptors in Italy. The authors performed an extensive six-year study to monitor the presence of these viruses, specifically targeting birds of prey due to their susceptibility and the critical role they play as sentinels for zoonotic diseases. The methodology involved capturing and sampling both migratory and resident raptors across various regions in Italy. The birds were subjected to RT-PCR tests to detect WNV and USUV. Notably, USUV was not detected in any samples, whereas WNV was found in several species, indicating the ongoing circulation of WNV in Italy. The study’s findings underline the significant role of avian species in monitoring the spread and emergence of zoonotic viruses. One of the strengths of the study is its comprehensive and longitudinal approach, allowing for an in-depth understanding of the virus’ dynamics over time. The data on the migratory behavior of the sampled birds also provided insights into potential routes of viral dissemination across regions. However, the study was limited by its focus on a specific group of birds, which may not represent the broader ecological and epidemiological context of WNV and USUV circulation. Overall, this research contributes valuable data to the field of zoonotic disease surveillance and highlights the importance of continuous monitoring and interdisciplinary approaches to managing the public health risks associated with viral pathogens in wildlife.

The fourth paper in this Special Issue, authored by Kasimov et al. [15], investigates the prevalence and genetic diversity of several pathogens in Australian wild birds. The researchers screened 486 wild bird samples from various locations in Southeast Queensland, focusing on five key pathogens: beak and feather disease virus (BFDV), *Chlamydiaceae*, avipoxvirus, columbid alphaherpesvirus 1 (CoAHV1), and psittacid alphaherpesvirus 1 (PsAHV1). Utilizing various qPCR assays, they identified BFDV in 33.54% of the birds, *Chlamydiaceae* in 20.16%, avipoxvirus in 9.47%, CoAHV1 in 8.85%, and PsAHV1 in 7.61%. This was the first detection of PsAHV1 in wild Australian birds, highlighting significant biosecurity concerns, especially for endangered parrot recovery programs. The study revealed that BFDV sequences clustered within two superclades affect both psittacine and non-psittacine species, although disease manifestation was observed only in psittacine species. The avipoxvirus sequences were identical to global reference strains, while PsAHV1 sequences were similar to those found in Brazilian psittacine species, suggesting potential intercontinental pathogen spread. This research underscores the high pathogen diversity in Australian avifauna and the potential risks of pathogen spillover into novel host species, which could have significant implications for wildlife conservation and public health. The findings emphasize the need for ongoing surveillance and comprehensive molecular studies in order to understand the ecological impacts and distribution of these pathogens better and to develop effective biosecurity measures. Overall, the study provides critical insights into the pathogen landscape of Australian wild birds and raises awareness of the potential biosecurity challenges posed by these diverse pathogens.

The final paper in this Special Issue, authored by Hoad et al. [16], presents a comprehensive analysis of the structural and biochemical interactions between the capsid protein of porcine adeno-associated virus (AAV) and the nuclear trafficking protein importin alpha (IMPα). This research identifies and characterizes a bipartite nuclear localization signal (NLS) within the porcine AAV capsid protein, which is crucial for nuclear import during viral infection. Utilizing techniques such as recombinant expression, purification, crystallization, and biochemical assays, the authors elucidate the binding affinities between the porcine AAV VP1 capsid protein and various importin isoforms. Key findings reveal that the porcine AAV capsid protein has a significantly higher binding affinity for IMPα isoforms than IMPβ, a strong interaction that is essential for the nuclear import of the viral genome, facilitated by the bipartite NLS within the capsid protein. A notable strength of this study is the comparative analysis of bipartite NLSs, which demonstrated markedly stronger binding to IMPα isoforms than monopartite NLSs, suggesting a more efficient nuclear import mechanism. Fluorescence polarization assays quantified these binding affinities, highlighting the specificity of the bipartite NLS for IMPα isoforms in the low nanomolar range. The study excels in its comprehensive structural analysis and the robust use of multiple experimental approaches to validate its findings. However, a critical limitation is the narrow focus on the porcine AAV capsid protein, which may not fully represent the diversity of AAV capsid proteins. The broader implications of this research for other AAV serotypes and potential applications in gene therapy warrant further exploration. Overall, this research significantly advances our understanding of viral nuclear import mechanisms and their role in AAV biology, although its applicability to other serotypes and contexts requires investigation.

Overall, this Special Issue highlights the critical need for interdisciplinary approaches to managing public health risks associated with viral pathogens in wildlife, emphasizing continuous monitoring, molecular studies, and comprehensive research in order to better understand and mitigate these emerging threats. Finally, I would like to thank the authors who contributed to this Special Issue and the reviewers for their fruitful input.
